# Can a Cerebral Congenital Anomaly Present in Adulthood?

**DOI:** 10.7759/cureus.31985

**Published:** 2022-11-28

**Authors:** Anas Mahmoud, Erinie Mekheal, Vibu Varghese, Patrick Michael

**Affiliations:** 1 Internal Medicine, St. Joseph’s University Medical Center, Paterson, USA; 2 Internal Medicine, St. Joseph's University Medical Center, Paterson, USA

**Keywords:** mri scan, ct scan, anti-psychotics, personality changes, pericardial effusion, altered mental status, neurocognitive impairment, ventricles, corpus callosum, colpocephaly

## Abstract

Colpocephaly, a congenital anomaly in the ventricles, is usually discovered early in infancy and rarely during adulthood. Partial or complete agenesis of the corpus callosum or Chiari malformations (developmental arrest of white matter formation in early fetal development) can lead to abnormal asymmetrical ventricular enlargement. Minimal literature about colpocephaly is available for clinicians, making diagnosis and treatment very challenging. Colpocephaly in adults is an infrequent condition, mostly found as an incidental finding with no neurological or cognitive impairment. Symptoms usually represent the affected lobe in the brain as our patient's visual hallucination may be attributed to the lesioned occipital horn. Differentiating from normal pressure hydrocephalus, representing new-onset dementia, can help avoid unnecessary procedures. Neurological and psychiatric consultation should be practiced to exclude other causes of neurological and cognitive impairment. While there is no definitive treatment for this condition, seizure prophylaxis has been helpful. Cognitive behavioral therapy, psychotherapy, and social skills training are recommended in some literature. Typical and atypical antipsychotics can control symptoms with uncertain efficacy.

## Introduction

Colpocephaly, a congenital abnormality rarely found in adults, was first diagnosed in a three-year-old boy with neurological manifestations (seizure, paralysis, and intellectual disability) in 1941 [1]. The main culprits include gene defects, anoxic brain injury, intrauterine infections, or other causes; however, many cases with no inciting factor were reported [2]. Diagnosis is often made by a CT or MRI scan of the brain, showing a disproportionate enlargement of the occipital horns with partial or full agenesis of the corpus callosum [3]. Idiopathic normal pressure hydrocephalus (NPH), a more common cause of acute change in personality with radiological characteristics of dilated ventricles, should be excluded as it will help avoid unnecessary procedures. A paucity of literature about colpocephaly in adults makes it very difficult to understand the disease's nature, course, management, and overall prognosis.

## Case presentation

**Figure 1:**
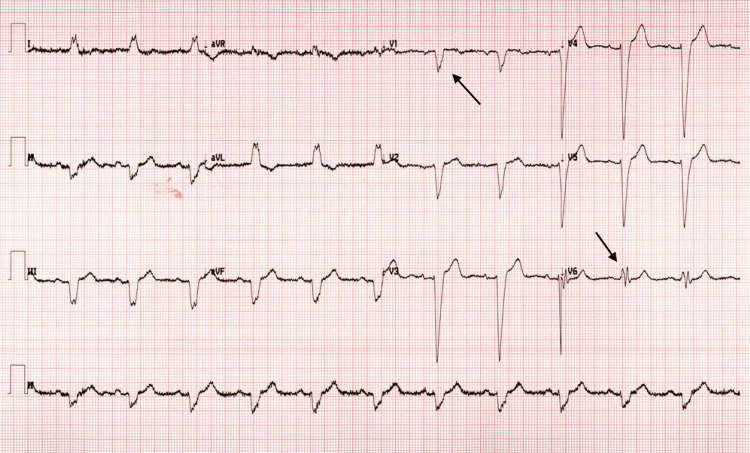
EKG showing left axis deviation and left bundle branch block QRS duration > 150 mm. A dominant S wave is seen in V1, and a broad, notched (‘M’-shaped) R wave is seen in V6, indicating a left bundle branch block. EKG: Electrocardiogram.

**Figure 2:**
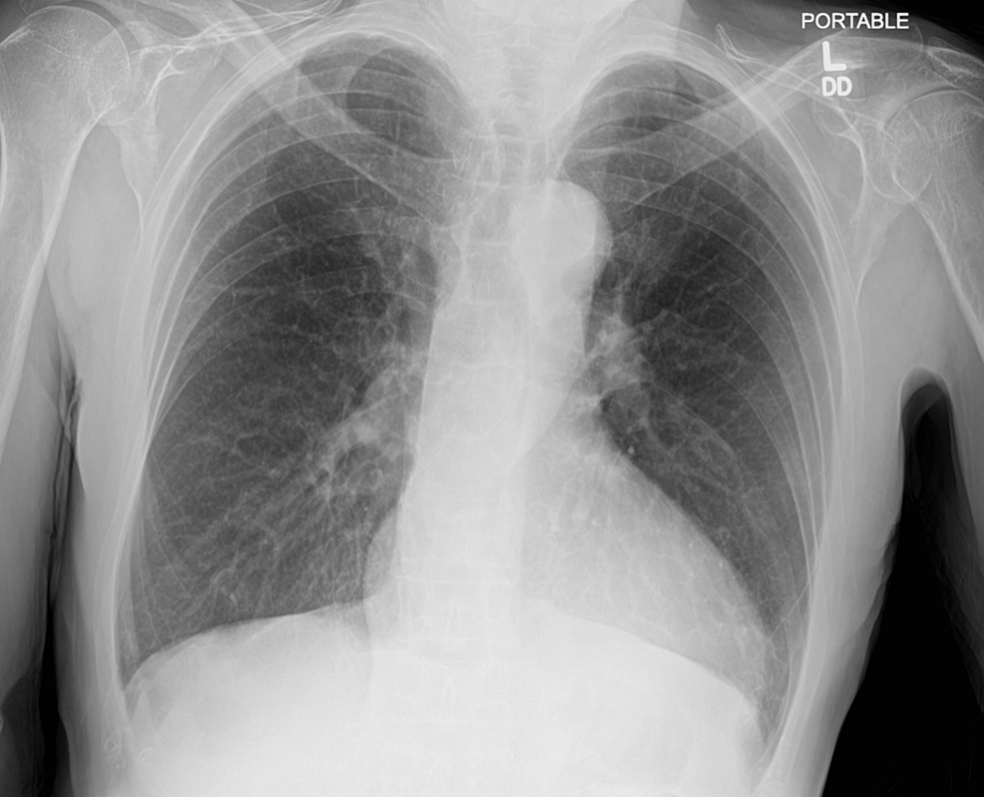
Chest x-ray with no apparent signs of effusion nor consolidation

The patient is a 62-year-old male with unknown past medical or psychiatric history who was brought to the emergency department by his neighbor for a new onset of a visual hallucination that started a couple of days earlier. In the emergency department, the patient was noted to be agitated and aggressive to the medical staff, waxing and waning his mental status. The patient reported seeing people in his house wearing hats, for which he called the police multiple times despite being informed that there were no people at his house. The patient endorsed that he has had hallucinations in the past and was hospitalized for them. He denied any recent history of fever, urinary problems, nausea, gait abnormality, or any other complaint. His niece, according to collateral history, indicated that the patient was completely normal during the last phone call two weeks earlier. His vitals are as follows: heart rate 78 bpm, blood pressure 150/67 mmHg, respiratory rate 16 bpm, and normal temperature 98 F. Labs were normal, and the physical exam showed normal neurological functions with no rigidity noticed. Medication reconciliation did not point out an offending medication. EKG (Figure 1) was suggestive of left axis deviation and left bundle branch block. Chest x-ray (Figure 2) did not show any consolidation, effusion, or pneumothorax.

**Figure 3:**
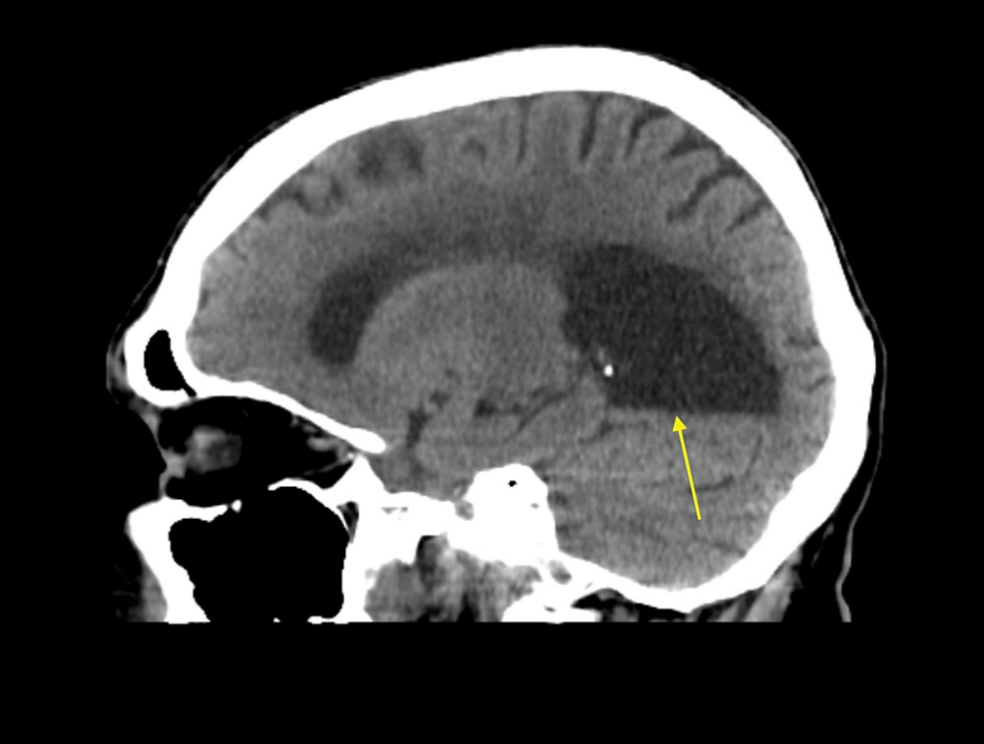
CT scan of the head: sagittal view. Asymmetric dilatation of the right occipital horn due to agenesis of corpus callosum resulting in colpocephaly.

**Figure 4:**
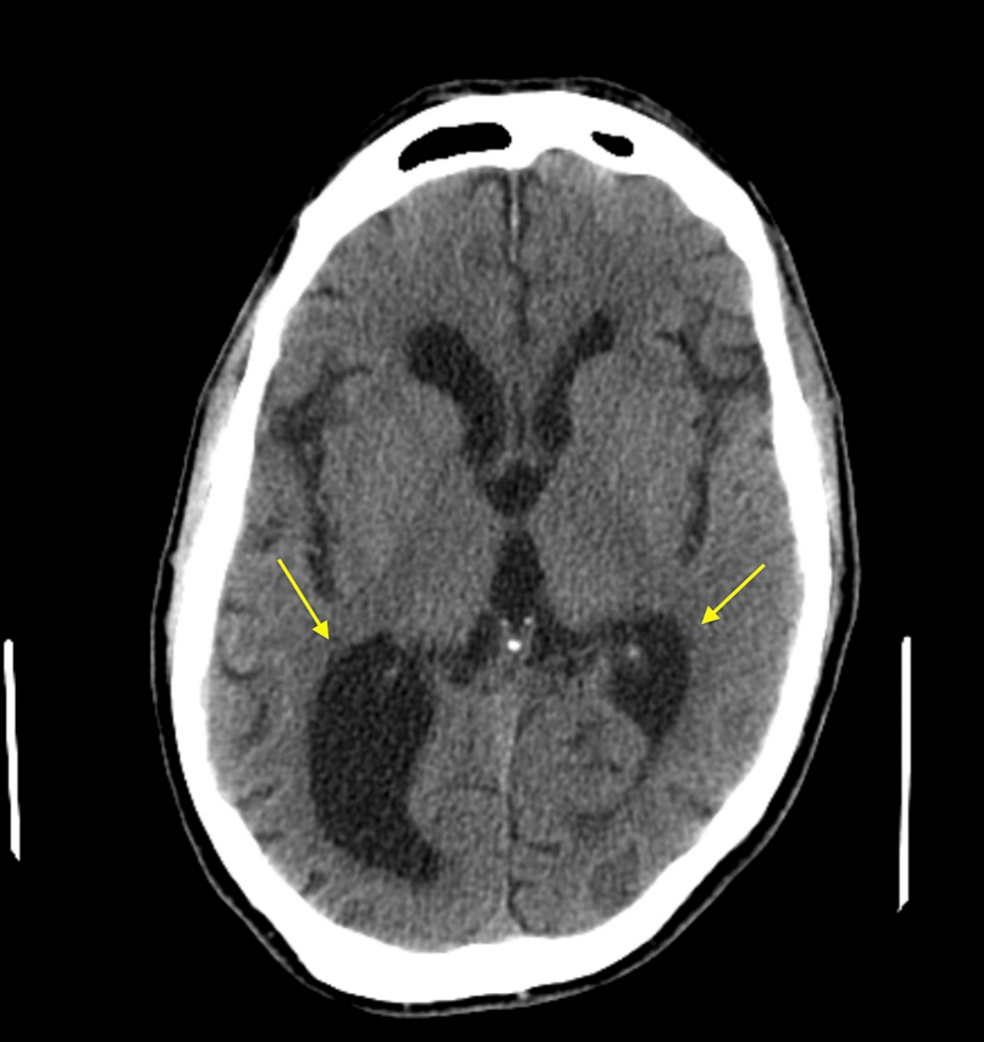
CT scan of the head: axial view. Asymmetric dilatation of the right occipital horn due to agenesis of corpus callosum resulting in colpocephaly.

CT scan of the head without contrast (Figures 3, 4) showed asymmetric dilatation of the right occipital horn, concerning colpocephaly.

The bladder scan was normal as well as the lumbar puncture. The echocardiogram showed pericardial effusion with tamponade, for which the patient had a subxiphoid pericardial window, which drained 500 ml of clear fluid. The patient was then admitted for delirium. Neurology and psychiatry teams followed the patient, and no identifiable cause for hallucination or personality change was found. The patient had recurrent pericardial effusion three weeks later; hence, a wide anterior pericardiectomy was done. The patient was started on olanzapine, which controlled both visual hallucination and agitation. The patient is still currently hospitalized, six months since the presentation, and he did not return to his baseline.

## Discussion

Hydrocephalus is an excessive amount of cerebrospinal fluid (CSF) within the cerebral ventricles, which leads to ventricular dilation [4]. It can be acute or chronic, which is reflected in the intracranial pressure, with acute hydrocephalus having elevated pressure. It can be congenital versus acquired and communicating versus obstructive. Communicating hydrocephalus causes include malformed formation, developmental or genetic mutation, achondroplasia, Chiari malformation, Dandy-Walker malformation, encephalocele, etc. Meanwhile, obstructive hydrocephalus could be due to hemorrhage, infection, arachnoid cysts, tumors, etc.

Hydrocephalus can present with headache, nausea, and vomiting due to increased intracranial pressure. It can also manifest as behavioral changes like irritability, aggressive behavior, or cognitive impairment, with unknown mechanisms. In neonates, psychomotor delay or gait dysfunction is well-observed. Establishing a relationship between psychosis and colpocephaly is not yet evidenced [5]. Hydrocephalus can lead to death if the enlarging ventricles compress vital brain stem functions.

A lumbar puncture can help when NPH or infections are highly suggested. Contrary to what one can expect, lumbar puncture is not necessary for establishing a diagnosis as it opposes the potential harm in patients with space-occupying lesions like an intracranial tumor or a brain abscess because of the risk of cerebral herniation. Therefore, CT and MRI should be performed first to avoid unnecessary risks.

In a case series [3], eight neonates were diagnosed with colpocephaly. Mental retardation was found in all of them, with a variety of motor abnormalities. In all cases, no definite cause was discovered; however, being born to a diabetic mother seemed to be a possible risk. While an error of morphogenesis was suggested in all cases, no definite management was concluded.

Colpocephaly in adults is a scarce condition, which is mostly found as an incidental finding with no neurological or cognitive impairment. To our knowledge, only three cases of adult-onset colpocephaly have been reported [1,6,7]. Esenwa and Leaf reported a 60-year-old woman presented with motor and cognitive impairment who was misdiagnosed as NPH, given the fact the patient presented with multiple falls and the rarity of colpocephaly in adults [1]. Cheong et al. reported a rare case of a 67-year-old woman with no neurological deficits who was found to have a typical meningioma in the posterior fossa and colpocephaly with agenesis of the corpus callosum; she underwent total resection of the tumor, followed by placement of the ventriculoperitoneal shunt [7]. Carter and Ffytche [8] reported a case of a 30-year-old man who presented with complex partial seizures and was found to have colpocephaly on an MRI scan of the brain, which revealed a structurally normal lateral neocortex of the right temporal lobe and a severe reduction in regional glucose consumption corresponding to the epileptic focus as seen in electroencephalogram (EEG). The three cases and our case demonstrate that mental retardation was not evident in adults with colpocephaly.

Symptoms were different in all reported cases, making it more challenging to establish a relationship between colpocephaly and the manifesting symptoms. Our patient's visual hallucination could be linked to the lesioned occipital horn suppressing the occipital lobe [8]. While there is no definitive treatment for this condition, seizure prophylaxis has been helpful. Cognitive behavioral therapy, psychotherapy, and social skills training are recommended in some literature [9] as Carter and Ffytche demonstrated in their transdiagnostic review of the relationship between visual hallucination and different diseases of dementia.

Typical and atypical antipsychotics can offer temporary relief for psychiatric symptoms; however, long-term management needs more literature. Neurological and psychiatric consultation can help exclude other causes of personality changes, cognitive impairment, and visual hallucination and help treat any reversible cause.

## Conclusions

Colpocephaly is a congenital anomaly characterized by a disproportionate dilation of the corpus callosum that very rarely can manifest in adulthood through different symptoms. Seizures, personality changes, or cognitive impairment have been reported in adults. Visual hallucination is common in different causes of dementia, but it can also be due to other medical conditions (substance abuse, psychosis, seizures, migraine, etc.). CT and MRI scans are the main standards in approaching hydrocephalus and helping prevent potential hazards of lumbar puncture in intracranial neoplasms. Colpocephaly is not well studied in adults, and our efforts to publish this case will help increase the available literature, which could benefit many patients.

## References

[1] Esenwa CC, Leaf DE (2013). Colpocephaly in adults. BMJ Case Rep.

[2] Puvabanditsin S, Garrow E, Ostrerov Y, Trucanu D, Ilic M, Cholenkeril JV (2006). Colpocephaly: a case report. Am J Perinatol.

[3] Garg BP (1982). Colpocephaly. An error of morphogenesis?. Arch Neurol.

[4] Paul LK, Brown WS, Adolphs R, Tyszka JM, Richards LJ, Mukherjee P, Sherr EH (2007). Agenesis of the corpus callosum: genetic, developmental and functional aspects of connectivity. Nat Rev Neurosci.

[5] Douzenis A, Rizos EN, Papadopoulou A, Papathanasiou M, Lykouras L (2010). Porencephaly and psychosis: a case report and review of the literature. BMC Psychiatry.

[6] Wunderlich G, Schlaug G, Jäncke L, Benecke R, Seitz RJ (1996). Adult-onset complex partial seizures as the presenting sign in colpocephaly: MRI and PET correlates. J Neuroimaging.

[7] Cheong JH, Kim CH, Yang MS, Kim JM (2012). Atypical meningioma in the posterior fossa associated with colpocephaly and agenesis of the corpus callosum. Acta Neurochir Suppl.

[8] Carter R, Ffytche DH (2015). On visual hallucinations and cortical networks: a trans-diagnostic review. J Neurol.

[9] Kosky KM, Phenis R, Kiselica AM (2022). Neuropsychological functioning in dysgenesis of the corpus callosum with colpocephaly. Appl Neuropsychol Adult.

